# Fe-S Clusters Emerging as Targets of Therapeutic Drugs

**DOI:** 10.1155/2017/3647657

**Published:** 2017-12-28

**Authors:** Laurence Vernis, Nadine El Banna, Dorothée Baïlle, Elie Hatem, Amélie Heneman, Meng-Er Huang

**Affiliations:** ^1^CNRS UMR 3348, Centre Universitaire, 91405 Orsay, France; ^2^Institut Curie, PSL Research University, UMR 3348, 91405 Orsay, France; ^3^Université Paris-Sud, Université Paris-Saclay, Centre Universitaire, UMR 3348, 91405 Orsay, France

## Abstract

Fe-S centers exhibit strong electronic plasticity, which is of importance for insuring fine redox tuning of protein biological properties. In accordance, Fe-S clusters are also highly sensitive to oxidation and can be very easily altered *in vivo* by different drugs, either directly or indirectly due to catabolic by-products, such as nitric oxide species (NOS) or reactive oxygen species (ROS). In case of metal ions, Fe-S cluster alteration might be the result of metal liganding to the coordinating sulfur atoms, as suggested for copper. Several drugs presented through this review are either capable of direct interaction with Fe-S clusters or of secondary Fe-S clusters alteration following ROS or NOS production. Reactions leading to Fe-S cluster disruption are also reported. Due to the recent interest and progress in Fe-S biology, it is very likely that an increasing number of drugs already used in clinics will emerge as molecules interfering with Fe-S centers in the near future. Targeting Fe-S centers could also become a promising strategy for drug development.

## 1. Introduction

**Figure 1:**
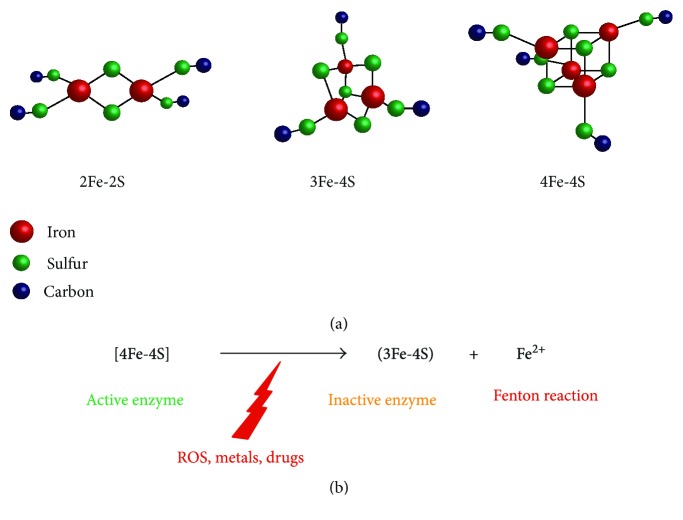
Most common iron-sulfur structures. (a) Most common Fe-S clusters associated with proteins contain 2, 3, or 4 iron atoms. Oxidation states of the cluster are variable and can be [2Fe-2S]^+^ or [2Fe-2S]^2+^, [3Fe-4S]^+^, [3Fe-4S]^0^, [3Fe-4S]^−^ or [3Fe-4S]^2−^, and [4Fe-4S]^3+^, [4Fe-4S]^2+^, [4Fe-4S]^+^, or [4Fe-4S]^0^. [3Fe-4S] clusters are most often considered as deriving from [4Fe-4S] clusters that have been oxidized by various cellular oxidants. Iron atoms are shown in red, sulfur atoms are shown in green, and carbon from cysteine residues are shown in dark blue. Coordination by histidine is not shown. (b) Conversion of [4Fe-4S] into [3Fe-4S] clusters is responsible for Fe^2+^ release and for enzyme inactivation. Fe^2+^ release might lead to Fenton reactions in the presence of hydrogen peroxide.

Iron-Sulfur (Fe-S) centers are small cofactors composed of iron and sulfur atoms that are bound to proteins. By exhibiting a high capacity of accepting or donating electrons, they allow efficient electron transport and subtle redox tuning of protein properties. They are mainly found under three forms, [2Fe-2S], [3Fe-4S], and [4Fe-4S], and are bound to proteins posttranslationally. In the majority of cases, the Fe ions are linked to sulfide ions and coordinated by cysteine and histidine ligands (see Figure 1). These ancient prosthetic groups allowed the appearance of fundamental processes during evolution, such as photosynthesis for example. Even though subsequent oxygenation of the Earth's atmosphere created a threat to Fe-S clusters that are typically oxygen-sensitive, it appears that an increasing number of eukaryotic proteins actually contain Fe-S centers. Fe-S proteins are present in all eukaryotic organelles and are involved in processes as diverse as electron transfer (e.g., respiratory chain complexes), enzymatic reactions (e.g., aconitase), and RNA and DNA metabolism (e.g., tRNA modification and activities of DNA polymerases *α*, *δ*, and *ε*, DNA primase, DNA2, and glycosylases [1]). It is now clear that Fe-S proteins are widely represented in all cellular essential processes and that altering Fe-S clusters by chemical approaches might have deleterious consequences for living cells.

**Figure 2:**
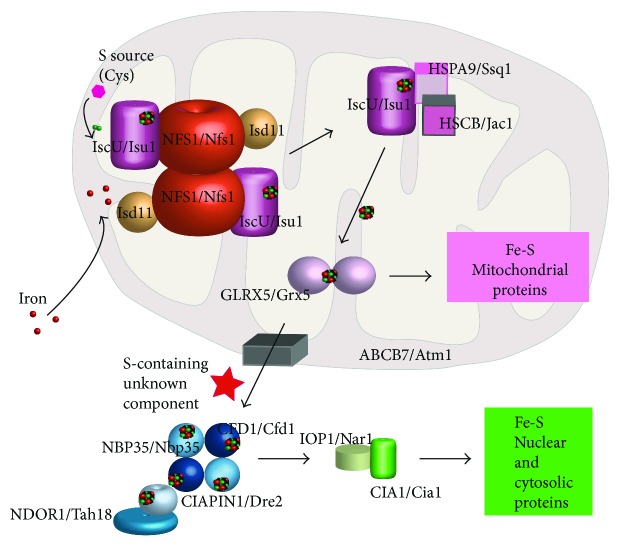
Schematic drawing of Fe-S cluster biosynthesis. Human/yeast proteins are indicated. Fe-S components are first synthesized in the mitochondria, and a yet unknown sulfur-containing component is exported into the cytosolic compartment. Further, Fe-S cluster biogenesis occurs, ultimately inserting clusters into recipient apoproteins.

In eukaryotic cells lacking plastids, Fe-S cluster biogenesis of all cellular Fe-S proteins is initiated by the mitochondrial iron-sulfur cluster (ISC) assembly machinery (Figure 2). Further maturation of extramitochondrial Fe-S proteins requires a yet unknown sulfur-containing compound being exported to the cytosol where the cytosolic Fe-S protein assembly (CIA) carries on with the process. Glutathione has been suggested to play a role in this transport process [2, 3] but this has not been demonstrated *in vivo* to date. Thanks to these highly regulated biosynthesis steps, Fe-S centers are present within different cell compartments, for example, nucleus, mitochondria, and cytosol. As a consequence, it is possible to target specifically one compartment or the other using Fe-S cluster destabilizing drugs with specific subcellular localization.

Due to their exceptional plasticity, Fe-S clusters can sense mild redox changes and act as cellular redox switches, thanks to redox or nuclearity changes, or even to degradation [4]. As so, biological functions associated to Fe-S-containing proteins can be modulated through oxidation, and these regulations have been particularly well studied in bacteria. For instance, oxygen reaction with the bacterial transcriptional regulator FNR (fumarate nitrate reductase regulator) provokes the conversion of a [4Fe-4S]^2+^ cluster into a [2Fe-2S]^2+^ cluster [5]. During this process, unstable [3Fe-4S]^1+^ species are generated and two Fe and two sulfide ions are released [6]. As a consequence, protein activity is inhibited or abolished by monomerizing the protein and preventing further DNA binding. Thus, Fe-S cluster oxidation provides a means to regulate protein activity in an oxygen-dependent manner.

Fe-S clusters on proteins are among the main targets of nitric oxide species (NOS), which are able to disrupt the cofactors [7]. Among them, nitric oxide (NO) is a highly reactive molecule, produced mainly by nitric oxide synthases. At a low concentration, NO is a signaling molecule implicated in numerous pathways, such as vasodilatation or response to infection, depending on subcellular concentrations [8]. In the bacteria *Mycobacterium tuberculosis*, for example, WhiB3 contains a [4Fe-4S] cluster which reacts specifically with NO, and more slowly with oxygen [9]. Noticeably, WhiB3 is considered as a major redox sensor. Its reactivity toward NO has major consequences for *Mycobacterium tuberculosis* physiology, as it controls redox homeostasis, lipid biosynthesis, and virulence [10]. Other studies showed that NO and peroxynitrite (ONOO^−^) directly attack Fe-S cluster in cytoplasmic aconitase (cyto-aconitase) in J774A.1 mouse macrophages. As a result, cyto-aconitase is converted into its apo form, the active iron regulatory protein 1 (IRP-1), together with iron release. IRP-2, which is also involved in iron homeostasis but does not coordinate any Fe-S cluster, is inactivated in the meantime by both NO and ONOO^−^. This deactivation/activation cycle of cyto-aconitase/IRP-1 by Fe-S cluster degradation is an example linking NOS to iron homeostasis and consequent inflammation regulation in macrophages [11]. As part of cellular regulation, cyto-aconitase Fe-S cluster alteration by NOS can be prevented in the presence of citrate [12]. Interestingly, NO was found to bind to human mitochondrial mitoNEET-related protein 2 (Miner2) [2Fe-2S] cluster but without disrupting the cluster, suggesting a new signaling mode for NO [13]. At higher concentrations, or if oxidant conditions persist, NOS and ROS can be definitely deleterious to Fe-S clusters. Oxygen, O_2_^•−^ and H_2_O_2_ can produce oxidative damage and convert [4Fe-4S]^2+^ clusters into [3Fe-4S]^1+^ and then into [2Fe-2S]^2+^ clusters that can ultimately be degraded. The apoprotein may lead to protein degradation, cell cycle arrest, and eventually cell death [14]. NO is also responsible for nitrosative damage, and noticeably thiol nitrosation. *In vitro* experiments indicated that Fe-S cluster nitrosylation reactions are complex and release several intermediates products, mainly iron nitrosyl chemical species [15–17].

Many drugs are able to produce NOS or ROS as a result of cellular catabolism and can logically alter Fe-S centers. Even though molecular mechanisms involved are not always fully understood yet, converging evidence indicates that Fe-S centers represent a privileged target of this category of drugs. An exhaustive list of drugs are presented throughout this review, which can either directly attack the Fe-S centers or produce reactive molecules that will alter Fe-S clusters. A synthetic view of this list is presented in Table 1, including possible mechanisms of action and therapeutical properties of the drugs.

## 2. Metals in the Cellular Environment Perturbing Fe-S Clusters

Sensitivity to metals is a characteristic of Fe-S groups. Copper is well known for being toxic to living cells, for example, and its antimicrobial properties have been used for a long time. Similarly, copper overloading leads to pathological situations in humans. On a cellular scale, it was shown that copper addition rapidly inactivates several Fe-S cluster-dependent enzymes, such as isopropylmalate dehydratase, and is responsible for copper toxicity [18]. Further, copper-induced Fe-S cluster alteration occurs without oxygen requirement, suggesting that copper damages result from liganding sulfur atoms that coordinate the clusters [18].

Aluminium is also known to be toxic to living organisms. Studies in *Pseudomonas fluorescens* demonstrated that aluminium actually perturbs Fe-S centers *in vivo* [19], based on analysis of spectrometric aconitase spectra in aluminium-stressed cells. Whether this perturbation is mediated by ROS or NOS or directly due to aluminium is not clear.

Cobalt is an essential heavy metal that can also be toxic in larger amounts. Cobalt toxicity has been well studied in bacteria, mainly *Escherichia coli* and *Salmonella enterica*, as cobalt was shown to interfere with Fe-S proteins metabolism [20]. Cobalt was proposed to compete with iron for sulfur assimilation and Fe-S cluster biogenesis, consequently compromising Fe-S cluster protein functions, including aconitase, succinate dehydrogenase, tRNA methylthiotransferase, and ferrichrome reductase [21, 22]. *In vitro* studies demonstrated that cobalt does not readily react with fully assembled Fe-S cluster, but with labile ones [21], underlining the importance of cell metabolism in mediating cobalt toxicity. Consistently, a moderate oxidative stress was evidenced within cells exposed to cobalt, which might take part in toxicity [23].

Finally, some but not all Fe-S centers have been shown to be direct targets of several other metals. [4Fe-4S] centers in various dehydratases from bacterial origin were all damaged by mild amounts of silver(I), mercury(II), cadmium(II), and zinc(II) [24]. Also, tellurite was shown to disrupt Fe-S clusters, in an ROS-dependent manner [25].

## 3. Fe-S Centers Are Targets of Drug-Induced ROS

Oxidative damage represents a major threat to cell survival, as explained in the introduction section. Because Fe-S clusters are particularly sensitive to ROS, they represent the first line of targets of oxidative stress. One example of these targets is the Fe-S cluster-containing protein RNase L inhibitor (Rli1). Rli1 is a highly conserved essential protein [26], involved in several key cellular process, such as ribosome biogenesis and recycling [27, 28], translation, initiation, and termination [29–31]. Most importantly, [4Fe-4S] cluster located in its N-terminus is crucial for protein function. Due to its central role in the cellular process described above, Rli1 is considered as a crucial target accounting for the inhibition of cell growth by ROS, and Rli1p dysfunction due to cluster alteration appears to be a deleterious outcome of oxidative stress [32]. Consequently, maintaining Rli1 function in aerobic organisms is of major importance, as Rli1's levels were shown to determine resistance to oxidative conditions. Interestingly, Rli1 was shown to be involved in resistance to copper. But, as opposed to isopropylmalate dehydratase (see previous paragraph; [18]), Rli1-dependent copper toxicity relies on the presence of oxygen; Rli1 clusters might actually be targeted by copper during transfer to apo-Rli1 in aerobic conditions [32].

Even though a highly debated question, killing bacteria through ROS production has been proposed to be a general mechanism for the three major classes of antibiotics, classified as follows: inhibition of DNA replication and repair (class I), protein mistranslation (class II), and inhibition of cell-wall turnover (class III) [33]. Bactericidal antibiotics induce hydroxyl radical formation via Fenton reaction due to cellular iron and NADH depletion. It is proposed that in case of cell exposure to bactericidal antibiotics, oxidative damage of Fe-S clusters is a key source of ferrous iron driving Fenton-mediated hydroxyl radical formation [34]. This was validated, for example, by the fact that mutants lacking the major Fe-S cluster biogenesis system ISC are tolerant to both antibiotics gentamicin (class II) and ampicillin (class III) [35]. Nevertheless, it is worth noting that antibiotics killing cells through ROS production is still a matter of debate [36, 37], and some authors proposed that bacteria are actually resistant in the absence of ISC not because they cannot synthesize Fe-S clusters but because they use the SUF (sulfur formation) system, an alternative Fe-S cluster biogenesis system, to build them [38]. In addition, fluoroquinolones fall into class I and are widely used thanks to their broad antimicrobial spectra, being active against both Gram-positive and Gram-negative bacteria. They are known to create DNA double-strand breaks, and thus inhibit bacterial growth or kill cells [39, 40]. Quinolones have also been demonstrated to act through ROS production by other authors [41–43]. In addition, it has been a long time knowledge that some amino acids can also inhibit bacterial growth, and among them, L-serine was found to exhibit the strongest effect [44, 45], due to the inhibition of homoserine dehydrogenase I, which is involved in the biosynthesis of threonine and isoleucine [46]. Combining L-serine together with two fluoroquinolones, ofloxacin or moxifloxacin, actually proves greater efficiency in killing bacteria, independent of growth phase. As previously identified [35, 43], this occurs through increasing the NAD^+^/NADH ratio, ROS production, and rapid Fe-S cluster disruption [47]. Whether Fe-S clusters are directly altered, in addition to their disruption by ROS, is not discussed. In a broader point of view, because resistance to antibiotics emerges significantly, which creates a threat to future generations, it is urgent to develop innovative antimicrobial strategies. Understanding the implication of altered Fe-S clusters by ROS-inducing antibiotics from different classes may help us decipher one hidden side of the resistance to antibiotics.

Fe-S cluster destabilization or/and alteration often lead to the apo form of the protein. As a consequence, the protein can switch to another function (case of the aconitase), be “repaired” as a new Fe-S center might be loaded, or be ultimately degraded (see [4] for review). *β*-Phenethyl isothiocyanate (PEITC) is a natural product with potent anticancer activity against human leukemia. PEITC administration leads to a rapid depletion of mitochondrial glutathione and an increased production of ROS and NOS [48]. Consequently, the Fe-S center of NADH dehydrogenase 3 from respiratory complex I is degraded, leading to significant suppression of mitochondrial respiration, which is at least partially responsible for PEITC anticancer activity. Also, combined treatment by dichloroacetate and aconitine-containing antiangiogenic agent BC1 proved significant antitumor activity against Ehrlich carcinoma [49]. Using this combination, substantial nitrosylation of Fe-S proteins was obtained. This effect occurred through a 2-fold reduction of Fe-S cluster cellular content and increased levels of Fe-S nitrosyl or dinitrosyl iron complexes (DNICs).

Beside ROS-producing drugs, specific NOS-producing drugs are being under development but are not as well characterized as ROS-producing drugs regarding potential Fe-S cluster-targeting properties [50]. It is also of importance that the half-life of NO is a function of oxygen concentration, making NO highly unstable in cells [51]. NO donors such as diazeniumdiolates (NONOates) have been manipulated and conjugated to other therapeutic molecules to improve their potential and have been tested in humans [52–54]. NO-donors are also being coupled to vehicles for improved targeting, but potential effects towards Fe-S clusters have not been precisely studied yet [55].

## 4. Fe-S Cluster-Targeting Drugs

**Figure 3:**
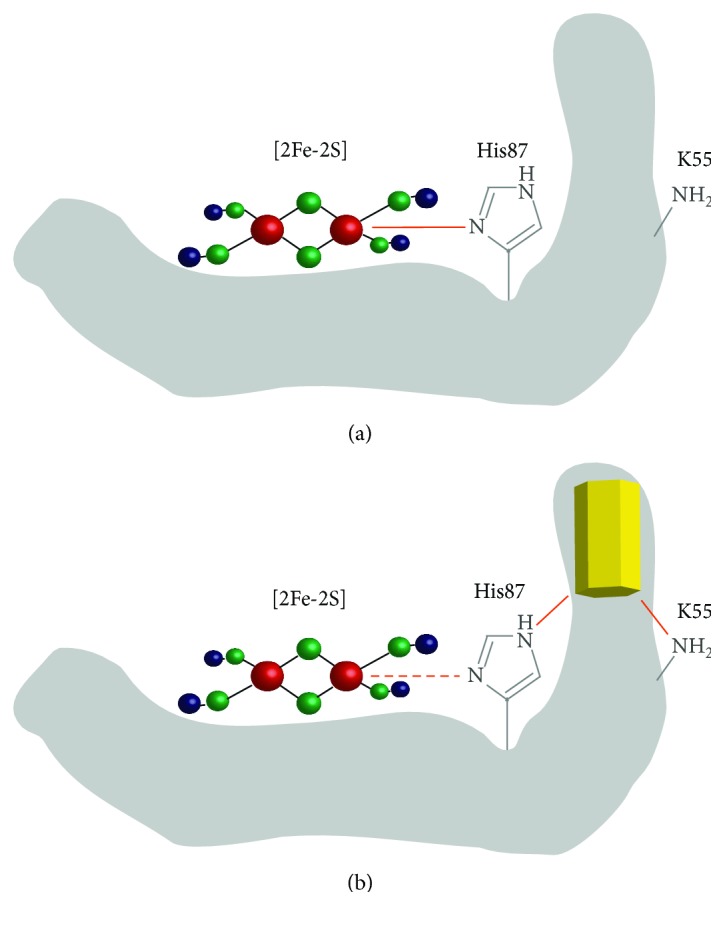
Cluvenone-derivative MAD-28 destabilizes mitoNEET [2Fe-2S] cluster. (a) Picture of mitoNEET protein (grey) with a [2Fe-2S] cluster coordinated by 3 cysteines (not shown) and one histidine (His87, shown in grey). The coordinating bond is shown in orange. (b) The influence of MAD-28 (yellow) binding to mitoNEET. MAD-28 set up bonds with both Lys55 and His87 and weakens the bond between Iron and His87, thus destabilizing the cluster.

Cluvenone (CLV) is a class of molecules with anticancer properties, targeting mitochondria and displaying good tumor selectivity [56]. The CLV derivative MAD-28 was reported to bind and destabilize two [2Fe-2S] proteins, mitochondrial mitoNEET and endoplasmic reticulum nutrient-deprivation autophagy factor-1, NAF-1 [57], both proteins being overexpressed in several cancer cell lines [58, 59]. MitoNEET is involved in the control of oxidative respiration, Fe-S cluster transfer, and electron transport. It is anchored to the outer mitochondrial membrane, with part of it located in the cytosolic compartment [60]. MitoNEET is involved in Fe-S protein repair, by reloading Fe-S clusters onto cytosolic proteins whose Fe-S clusters have been removed or altered [61]. MitoNEET forms a dimer with one [2Fe-2S] cluster per monomer, strikingly coordinated by three cysteines and one histidine, His87 [62], different from 4-Cys or 2-Cys/2-His ligation in ferredoxins or Rieske centers [63]. In the case of NAF-1, the unique 3Cys-1His cluster is now thought to be involved in promoting rapid tumor growth [64]. Because MDA-28 breaks the coordinative bond between the His ligand and the cluster's Fe of mitoNEET and NAF-1, it destabilizes the cluster (Figure 3). As a consequence, MDA-28 strongly inhibits cell proliferation and reveals high specificity in selective killing of cancer cells. Therefore, MAD-28 is being considered as a new potent anticancer agent, and mitoNEET and NAF-1 as a novel family of anticancer targets [57, 64–66].

MitoNEET has been also identified recently as a target for the thiazolidinedione (TZD) class of diabetes drugs, including pioglitazone [67]. Drugs from the TZD class actually bind mitoNEET and act by stabilizing the oxidized state of the cluster, which is otherwise most likely in a reduced state due to cytosolic reducing environment [63]. This stabilization may involve His87, as a His87Cys mutation mimics pioglitazone exposure, by counteracting cluster lability [68, 69]. His87 was actually proposed to be critical for communication with the Fe-S center of mitoNEET [63]. It also prevents the [2Fe-2S] cluster release [70], thus interfering with mitoNEET Fe-S cluster rebuilding activity. Similar effects have been observed on NAF-1 [71].

In addition to the abovementioned drugs, other molecules present naturally in the cell have been reported to interact and destabilize mitoNEET Fe-S clusters. It is the case of reduced nicotinamide adenine dinucleotide phosphate (NADPH) that binds to mitoNEET and destabilizes the cluster, resulting in Fe-S cluster decomposition, as NADPH binding facilitates Fe-S cluster release from the protein [72]. It is interesting to notice that increased NADPH levels in cancer cells correlate with an increase in mitoNEET levels, which could be due to an adaptive cellular response to Fe-S cluster destabilization. Elevated NADPH pool is of importance in cancer cells as they provide reducing equivalent required for high levels of nucleotide, protein, and fatty acid found in proliferating cells and for counteracting oxidative damage due to increased ROS production. At the molecular level, mitoNEET residues Lys55 and His58 are involved in NADPH binding on one subunit, which might in turn compromise the interaction with His87 and Arg73 from the other subunit, underlying the key role of His87 as in the case of pioglitazone (see above). NADPH binding to mitoNEET also inhibits transferring [2Fe-2S] clusters from mitoNEET to apo-acceptor proteins *in vitro* at physiological NADPH concentrations, suggesting that NADPH might control mitoNEET [2Fe-2S] cluster levels and its ability to transfer [2Fe-2S] clusters to cytosolic or mitochondrial partners [73]. Based on the impact of NADPH on mitoNEET, it is tempting to suggest that modulation of cytosolic NADPH pool is a good strategy for antitumor therapy in combination with other anticancer drugs [74].

Cytochrome c is a hemoprotein residing within the intermembrane space of mitochondria, whose role in activating programmed cell death apoptosis has been well established. Cytochrome c does not contain any Fe-S center per se, but still, the heme iron is coordinated to the sulfur atom of Met^80^ (Fe-S (Met^80^) bond). This bond plays a major role in apoptosis activation by different drugs; as so, it is worth mentioning it in this review as a good illustration of Fe-S bond disruption and consequences *in vivo*. In living cells, cytochrome c participates in electron shuttling between respiratory complexes III and IV. When interacting with cardiolipin, partial unfolding of cytochrome c occurs and allows switching to a peroxidase, then leading to apoptosis. Analogs of vitamin E, *α*-tocopherol succinate (*α*-TOS), and *α*-tocopherol phosphate (*α*-TOP) have been found to play similar roles in the interaction with cardiolipin in that they disrupt the Fe-S Met^80^ bond associated with unfolding of cytochrome c. This mechanism may underlie anticancer properties of vitamin E derivatives, otherwise considered as antioxidants [75], through promoting the execution of the apoptotic program.

## 5. Defects in Fe-S Metabolism Sensitize Cells to Drugs

As Fe-S centers are essential for cell viability, it is likely that Fe-S cluster-targeting drugs combined to intrinsic defects in Fe-S cluster biogenesis can have additive or synergistic effects. Indeed, defects in Fe-S metabolism have been reported to sensitize cells to drugs. The fungal pathogen *Cryptococcus neoformans* is responsible for meningitis in immunocompromised individuals. A mutation in the ferroxidase Cfo1 provokes reduced iron uptake and iron homeostasis perturbations, as well as mitochondrial respiration and Fe-S cluster biogenesis alterations. In addition, this mutant shows a marked susceptibility to the azole antifungal fluconazole, a situation which can be mimicked when treating fungal cells with the respiration inhibitor diphenyleneiodonium [76]. Overall, this work suggests that iron homeostasis and decreased cellular Fe-S cluster synthesis play a key role in antifungal susceptibility.

CTBT (7-chlorotetrazolo[5,1-c]benzo[1,2,4]triazine) is known to enhance the activity of several antifungal agents [77]. Further analysis of CTBT mode of action identified that this compound causes intracellular superoxide production and oxidative stress [78], consistent with rapid activation of oxidative stress response pathway under the control of Yap1 and Cin5 and thus likely altering Fe-S centers *in vivo*. By screening mutant collection for mutants sensitive to CTBT, authors indeed identified, among others, *isa1* and *isa2* mutants with decreased cytosolic and mitochondrial Fe-S cluster biogenesis [78], indicating that alteration of Fe-S clusters by intracellular acute ROS production plays a synergistic role with intrinsically diminished Fe-S cluster biogenesis.

Hydroxyurea (HU) is an anciently synthesized therapeutic agent used in clinics to mainly treat sickle cell disease and is known to slow down DNA replication *in vivo* by inhibiting ribonucleotide reductase, a multimeric enzyme responsible for dNTP biosynthesis. In a recent study [79], HU was found producing ROS that are deleterious for cellular Fe-S centers, thus rendering mutants exhibiting reduced Fe-S cluster biogenesis particularly sensitive to HU. In this example again, yeast mutants with defective cytosolic Fe-S cluster biogenesis show high sensitivity to HU, illustrating that synergistic effects on Fe-S cluster alteration resulted from both ROS production and intrinsic decreased Fe-S cluster biogenesis [79].

## 6. Drugs That Alter Fe-S Biosynthesis Pathway and Fe-S Cluster Level Sensing

In the course of an interesting work, trying to circumvent antimicrobial resistance in *Staphylococcus aureus* strains, a new molecule named “‘882” was identified, whose toxicity to bacterial strains relies on the inhibition of the Fe-S cluster synthesizing complex SUF [80]. ‘882 was shown to physically interact with the SUF Fe-S cluster biogenesis machinery (SUFC, B, D, and S), and consequently, activity of the Fe-S cluster-dependent enzyme aconitase was decreased in presence of ‘882. ‘882 thus has pleiotropic effects on the Fe-S cluster biosynthesis machinery.

IscR is a global transcription regulator containing a [2Fe-2S] cluster in bacteria, which represses transcription of the operon containing its own gene and the iscSUA-hscBA-fdx genes, whose products are involved in Fe-S cluster biogenesis [81]. IscR also participates in the regulation by oxygen of several promoters controlling the expression of anaerobic Fe-S proteins [82]. In-depth characterization of the [2Fe-2S] cluster in IscR showed an atypical coordination of the cluster by three cysteines and one histidine, suggesting that IscR might be a sensor of cellular Fe-S cluster status [83]. This idea was also further taken up by others, elaborating that IscR might modulate intracellular iron homeostasis by directly repressing or activating the transcription of genes affecting these pathways [84]. Within the same idea, WhiB7 in *Mycobacterium tuberculosis* is a transcriptional regulator containing four cysteines that coordinate a redox-sensitive Fe-S cluster or form disulfide bonds [85, 86]. WhiB7 is dependent upon Fe-S for folding as cysteine mutations increase Fe-S release and WhiB7 instability [87]. Interestingly, WhiB7 expression responds to several antibiotics and is also synergistically enhanced by the presence of a reducing drug in the medium [85]. It is thus a possibility that alteration of Fe-S by drugs within the cells are directly sensed; nevertheless, no such sensor has been identified until now.

## 7. Cellular Respiration Modulates Fe-S Cluster Sensitivity to Drugs

Strikingly, drug toxicity has often been shown to be enhanced or modulated by cellular respiration. Recent work in yeast demonstrated also that Fe-S clusters are targets of the antimalarial drug primaquine [88]. Exposure of yeast cells to primaquine further decreased the activity of aconitase and Rli1, two proteins relying on Fe-S clusters for activity as described before, and thus are sensitive to oxidative damage. The authors proposed that ROS-labile Fe-S groups might be the primary target of primaquine *in vivo*. Moreover, primaquine also alters primase activity *in vitro*, suggesting likely a direct interaction of the drug with labile Fe-S clusters. In addition, authors also identified that the growth inhibitory effect of primaquine relies on respiration and that ROS produced by respiration play a major role in this process. Actually, while the sensitivity of yeast cells to the antimalarial drug primaquine was observed only when cells grew using respiration, the drug had no or little effect on cells undergoing fermentation, indicating that respiratory activity enhances the deleterious effect of primaquine [88]. It is also possible that primaquine reacts with ROS endogenously produced during respiration, which would then generate an even more toxic compound.

Nevertheless, Fe-S cluster-containing proteins such as Nar1 (nuclear architecture related 1), an essential subunit of the cytosolic Fe-S protein assembly machinery, and Rli1 are also essential during fermentative growth in the absence of respiration. It is thus a possibility that Fe-S clusters from the respiratory chain proteins are preferentially targeted for degradation, as compared with Fe-S proteins from other cellular compartments. This hypothesis is actually supported by results obtained with the anticancer drug PEITC. PEITC induces significant suppression of mitochondrial respiration due to the favored degradation of the Fe-S center from NADH dehydrogenase 3 within respiratory complex I [48]. Decreasing respiration may mostly account for PEITC anticancer property.

Several independent studies have identified that increased respiratory metabolism renders cells more sensitive to several drugs, such as anticancer biguanide drugs, that inhibit mitochondrial complex I [89]. Also, triple-negative breast cancer cells are specific cancer cells that do not respond either to hormonal therapy or to HER2-targeted therapy, and in the meantime, they exhibit profound metabolic changes, with decreased mitochondrial respiration and increased glycolysis [90]. These changes are often suggested as being causative in the resistance to different treatments [91], even though most of the drugs in question have not been studied in the light of Fe-S metabolism yet. As evoked before, it is tempting to speculate that Fe-S proteins from the respiratory chain might be a privileged target for numerous therapeutic drugs, linking decreased respiratory activity to drug resistance.

## 8. Targeting Fe-S Centers Might Be a Promising Strategy

There is now interest in identifying new pathways that might be targeted by newly developed drugs, as illustrated by the alarming increase in the number not only of bacterial pathogen strains that are resistant/tolerant to antibiotics [92] but also of other diseases such as cancers with unsuccessful treatments until now. In this perspective, targeting Fe-S clusters has been proposed as a strategy to fight some pathogens in humans.

The enzymes of the SUF pathway for example are essential for bacterial pathogens but are significantly distant from proteins of eukaryotic origin. For these reasons, SUF enzymes have been suggested to be attractive candidates in the search of new drug targets [93]. *Mycobacterium tuberculosis* is responsible for tuberculosis, a major, still uncontrolled threat to global health. Taking advantage of severe phenotypes induced by disrupting iron homeostasis in this organism, targeting Fe-S clusters has been considered as an interesting option [94]. The essential adenosine 5′-phosphosulfate reductase (APR) is a [4Fe-4S]-containing enzyme in *M. tuberculosis*. Several adenosine analogs were developed and selected for the presence of Fe and S binding groups such as thiols or carboxylic and hydroxamic acids, providing an improved solid-phase method as an approach for the development of a new class of APR inhibitors [95].

As previously mentioned, ‘882 is a recently developed therapeutic molecule against *Staphylococcus aureus* that demonstrates how Fe-S cluster assembly pathway modulation by small molecules is an interesting option in controlling pathogens and may guide the development of new compounds that target this essential pathway [80].

Recent work evidenced the presence of Fe-S clusters within the Merkel cell polyomavirus (MCPyV) small T (sT) antigen, which plays the role of an oncogenic driver in Merkel cell carcinoma (MCC) [96]. MCPyV sT translocates to nuclear foci containing actively replicating viral DNA, supporting a direct role for sT in promoting viral replication. MCPyV sT coordinates a [2Fe-2S] and a [4Fe-4S] cluster, and mutations in the coordinating cysteines abolish its capacity to stimulate viral replication. This discovery supports the idea that targeting the coordination of MCPyV sT might be of therapeutic interest.

ROS-modulating strategies have been proposed in combination with other drugs to enhance therapeutic efficacy. The rational hypothesis is to take advantage of chronically increased oxidative stress levels within cells, leading to preferential killing of those cells in the presence of an additional ROS bolus, typically bacteria in the course of infection, or cancer cells [97, 98]. Because Fe-S clusters are typically ROS-sensitive, it is likely that ROS-modulating approaches combined with Fe-S cluster targeting compounds might be of great interest.

Finally, Fe-S cluster targeting strategies based on Fe-S degradation and/or disintegration following drug treatment may have a static effect, inducing metabolic pausing in pathogens [99] because several Fe-S clusters have been described as “repairable” [100–102] and because Fe-S biogenesis might be impaired only transiently. Fe-S cluster targeting drugs might thus not always lead to rapid cell death. This aspect will be of importance when considering combining Fe-S cluster targeting with other cell killing modes.

## Figures and Tables

**Table 1:** Therapeutic drugs interacting with Fe-S clusters.

Drug	Therapeutical property	Therapeutic indication	Fe-S cluster-containing target	Mechanism	Reference
Hydroxyurea	Antiproliferative	Sickle cell disease, leukemia, polycythemia vera, other cancers	Leu1	ROS-mediated	[79]
Primaquine	Antiparasite	Malaria	Rli1, aconitase	Fe-S cluster interaction and ROS-mediated	[88]
MAD-28 (cluvenone derivative)	Antiproliferative	Cancer	MitoNEET, NAF-1	Fe-S cluster destabilization	[57]
Cluvenone	Proapoptotic	Acute lymphoblastic leukemia	MitoNEET, NAF-1	Fe-S cluster stabilization	[56, 57]
Pioglitazone (thiazolidinedione family)	Antidiabetes insulin sensitizer	Diabetes	MitoNEET, NAF-1	Fe-S cluster stabilization	[67, 70, 71]
‘882	Antimicrobial		SUF machinery	Binding to Fe-S cluster biogenesis machinery	[80]
Antibiotics	Antimicrobial	Bacterial infections		ROS-mediated (still a matter of debate)	[35, 41–43, 47]
*β*-Phenethyl isothiocyanate (PEITC)	Antiproliferative	Leukemia	NADH dehydrogenase 3 (respiratory complex I)	ROS-mediated	[48]
BC1	Antiangiogenic, antitumor	Ehrlich carcinoma		ROS-mediated	[49]
